# Evaluation of Sex-Specific Gene Expression in Archived Dried Blood Spots (DBS)

**DOI:** 10.3390/ijms13089599

**Published:** 2012-08-02

**Authors:** James H. Resau, Nhan T. Ho, Karl Dykema, Matthew S. Faber, Julia V. Busik, Radoslav Z. Nickolov, Kyle A. Furge, Nigel Paneth, Scott Jewell, Sok Kean Khoo

**Affiliations:** 1 Program of Biospecimen Science, Van Andel Research Institute, Grand Rapids, MI 49503, USA; E-Mails: james.resau@vai.org (J.H.R.); scott.jewell@vai.org (S.J.); 2 Department of Epidemiology & Biostatistics, College of Human Medicine, Michigan State University, East Lansing, MI 48823, USA; E-Mails: nho@epi.msu.edu (N.T.H.); paneth@epi.msu.edu (N.P.); 3 Laboratory of Computational Biology, Van Andel Research Institute, Grand Rapids, MI 49503, USA; E-Mails: karl.dykema@vai.org (K.D.); kyle.furge@vai.org (K.A.F.); 4 Department of Physiology, Michigan State University, East Lansing, MI 48824, USA; E-Mails: fabermat71686@gmail.com (M.S.F.); busik@msu.edu (J.V.B.); 5 Department of Mathematics & Computer Science, Fayetteville State University, Fayetteville, NC 28301, USA; E-Mail: rnickolov@uncfsu.edu; 6 Department of Pediatrics & Human Development, College of Human Medicine, Michigan State University, East Lansing, MI 48823, USA; 7 Laboratory of Microarray Technology, Van Andel Research Institute, Grand Rapids, MI 49503, USA

**Keywords:** archived dried blood spots (DBS), sex-specific, gene expression, molecular genetic profiling, microarray

## Abstract

Screening newborns for treatable serious conditions is mandated in all US states and many other countries. After screening, Guthrie cards with residual blood (whole spots or portions of spots) are typically stored at ambient temperature in many facilities. The potential of archived dried blood spots (DBS) for at-birth molecular studies in epidemiological and clinical research is substantial. However, it is also challenging as analytes from DBS may be degraded due to preparation and storage conditions. We previously reported an improved assay for obtaining global RNA gene expression from blood spots. Here, we evaluated sex-specific gene expression and its preservation in DBS using oligonucleotide microarray technology. We found X inactivation-specific transcript (*XIST*), lysine-specific demethylase 5D (*KDM5D*) (also known as selected cDNA on Y, homolog of mouse (*SMCY*)), uncharacterized LOC729444 (*LOC729444*), and testis-specific transcript, Y-linked 21 (*TTTY21*) to be differentially-expressed by sex of the newborn. Our finding that trait-specific RNA gene expression is preserved in unfrozen DBS, demonstrates the technical feasibility of performing molecular genetic profiling using such samples. With millions of DBS potentially available for research, we see new opportunities in using newborn molecular gene expression to better understand molecular pathogenesis of perinatal diseases.

## 1. Introduction

Newborn screening is a mandated and routine health care program in many countries such as the United States, Japan, and most European countries [1]. Blood is collected from a heel prick 24–48 h after birth, placed on a Guthrie card and air-dried before submission to laboratories for testing. Quantitative analytical assays for newborn screening range from simple approaches such as colorimetric assays to advanced technologies such as liquid chromatography-tandem mass spectrometry [2]. After newborn screening, unused blood spots and/or portions of blood spots remain, and these are either discarded or archived, depending upon prevailing law and practice. Archived dried blood spots (DBS) are kept under varied storage conditions (e.g., frozen or unfrozen) and for varied durations of time [1]. Some DBS have been kept since the beginning of newborn screening programs 30 years ago, and collections of DBS now number several millions in some US states. DBS represent a unique retrospective research material that comprehensively yet inexpensively captures virtually 100% of the newborn population, making it an ideal resource for molecular epidemiological studies.

The use of DNA and RNA as genomic templates for advanced molecular applications usually involves high-quality, non-degraded isolates from fresh and “unfixed” samples. However, when clinical material is the source, investigators must contend with imperfections in the sample stemming from fixation in surgical pathology paraffin blocks or use of other preservation methods, uncertain collection procedures, and in the case of many DBS samples, long-term storage at ambient temperatures. To maximize the public health value of the advanced molecular tools now available to examine such clinical material, it is important to develop, establish, and validate new molecular assays on such samples, and not only with ideal samples generated in well-controlled laboratory experiments. A particular challenge in relation to the use of DBS in molecular genomic studies is the presumed low quantity and degraded quality of nucleic acids that can be isolated from these samples. Nevertheless, DNA from DBS has been successfully isolated for molecular assays such as PCR, single nucleotide polymorphisms (SNPs) genotyping, genome-wide scanning and, direct sequencing [3–10]. RNA, however, is less stable under certain circumstances, degrades more easily than DNA, and can be more challenging to isolate from dried blood spots [11]. Nonetheless, several recent studies have detected RNA from viruses and naïve T-cells of dried blood spots using quantitative real-time PCR assays [12,13]. Our research team has also established molecular protocols for detecting global RNA gene expression from blood spot samples, both fresh and archived, using microarray technology [14,15].

Although improved assays have been established to attain global gene expression from DBS, the feasibility of acquiring differentially-expressed genes from one trait to another (e.g., diseased *vs*. healthy controls) to better understand their biological functions has yet to be proven. Here, we evaluate the fidelity of sex-specific RNA expression in DBS to show the feasibility of acquiring differentially-expressed genes from 75 males and 31 females.

## 2. Results and Discussion

### 2.1. Differentially-Expressed Genes can Be Determined in DBS

**Figure 1:**
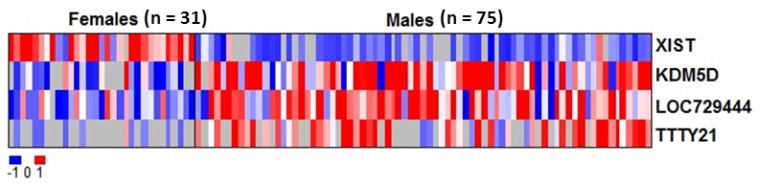
A heatmap showing four differentially-expressed genes between males (*n* = 75) and females (*n* = 31) which were statistically significant. Red indicates over-expressed transcripts, blue represents under-expressed transcripts, and grey represents absence of data due to unmet filtering criteria.

To address the concern of whether the overall RNA gene expression can be sufficiently preserved in DBS to answer significant biological questions, we investigated the gene expression of males *vs*. females acquired from DBS using microarray technology. We identified four statistically significant differentially-expressed genes between groups of male and female subjects. These genes were X inactivation-specific transcript (*XIST*), lysine-specific demethylase 5D (*KDM5D*) which is also known as selected cDNA on Y, homolog of mouse (*SMCY*), uncharacterized LOC729444 (*LOC729444*), and testis-specific transcript, Y-linked 21 (*TTTY21*) (Table 1). All genes were sex-related. The expression of *XIST* was lower in males than in females. *KDM5D* and *LOC729444* were over-expressed in males compared to females while *TTTY21* was under-expressed in females compared to males (Figure 1). Although other sex-related transcripts such as testis determining factor (*TDF*) could be detected, the gene expression was low in general and there was no significant difference between males and females.

### 2.2. RNAs Related to Sex Are Preserved in DBS

**Figure 2:**
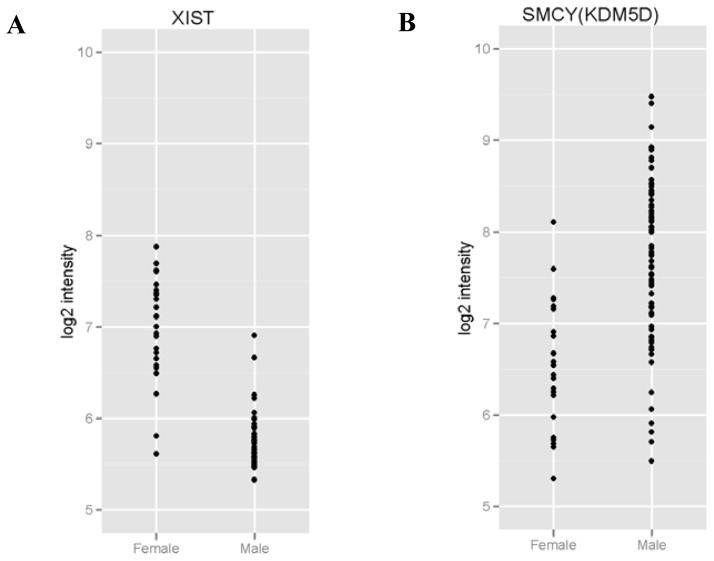
(**A**) Evidence of female-specific gene expression preservation in dried blood spots (DBS) by dot plot analysis of *XIST. XIST* gene probe intensities showed differentiation and preservation of its RNA gene expression in males *vs*. females; (**B**) Evidence of male-specific gene expression preservation in DBS by dot plot analysis of *KDM5D. KDM5D* gene probe intensities showed differentiation and preservation of its RNA gene expression in males *vs.* females.

Of the 4 genes that we identified, *LOC729444* is an unknown gene while *TTTY21* is a non-protein coding transcript. On the other hand, *XIST* and *KDM5D* are well-documented genes related to sex traits. Thus, we generated scatter plots using gene expression data (normalized probe intensities) of *XIST* and *KDM5D* to investigate whether RNAs related to sex is preserved. Our results demonstrated that the expression of *XIST* were generally higher in females than in males and vice versa for *KDM5D*. We confirmed that sexes can be distinguished and gender status is preserved in DBS (Figure 2A,B).

### 2.3. XIST Expression can Be Validated Using qRT-PCR

**Figure 3:**
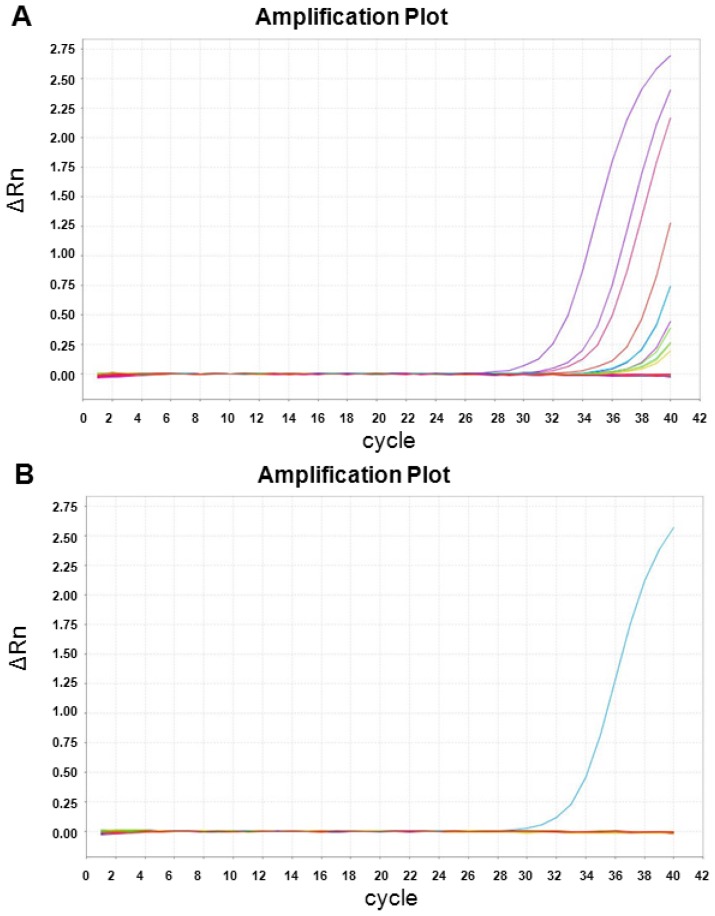
qRT-PCR validation of *XIST* gene expression in (**A**) females and (**B**) males. *Rn* is the fluorescence reporter dye signal normalized to the fluorescence signal of ROX dye. Δ*Rn* is *Rn* minus the baseline. Each curve represents the amplification linear plot of each sample.

We validated the RNA gene expression of *XIST*, the most significantly differentially-expressed sex-specific gene, using qRT-PCR. In the microarray data, expression signal of *XIST* was weak in both sexes, but the expression signal in males was significantly lower than in females. The qRT-PCR result reflected this weak signal, in that we found a detectable *XIST* signal in only 12 of 31 females in our samples (38.7%) (Figure 3A). However, detection of *XIST* by qRT-PCR was found only once in 75 male samples (1.3%) (Figure 3B) (*p* < 2.95 × 10^−6^; Fisher’s exact test). Our observation confirmed the findings of microarray data that RNA gene expression of *XIST* is overall lower in males than in females.

### 2.4. Discussion

It is a fact that high quality RNA is more difficult to acquire and store as it is more easily compromised when compared with DNA in human clinical samples. Therefore, it is logical to ask if any usable RNA can be obtained from DBS which are usually kept at ambient temperature. With the advancement of molecular tools and technologies, we recently established a technical protocol for using unique amplification systems to amplify non-poly A transcripts from compromised RNA and hybridizing them onto microarrays (with 60-mer length oligonucleotide probes) to obtain gene expression data from blood spot samples [15]. However, there remains the concern of whether the overall RNA gene expression can be sufficiently preserved in DBS to answer significant biological questions. To address this concern, we investigated the gene expression of 75 males and 31 females from DBS aged 2.5–15.6 years using microarray technology and evaluated their differentially-expressed genes. We identified 4 differentially-expressed genes which were statistically significant between males and females: *XIST*, *KDM5D*, *LOC729444*, and *TTTY21*. All four genes are sex-related.

*XIST*, which is mapped on Xq13.2, is one of those genes involved in X chromosome inactivation [16]. The X-inactivation center (*XIC*) regulates X-inactivation by counting the number of X chromosomes and contains *XIST* which localizes exclusively in the inactive X chromosome [17]. Thus, *XIST* is only expressed in the inactive chromosome X of a female. Our results are in concordance with the above statement, showing significantly lower *XIST* expression (*p* < 1.6 × 10^−16^) in male than in female DBS samples. In addition, its gene expression from qRT-PCR was in concordance with the microarray data. *KDM5D*, also known as selected cDNA on Y, homolog of mouse (*SMCY*), is first isolated in the short arm of the mouse Y chromosome [18]. In mouse, its expression can be detected as early as in the 2-cell stage during blastocyst development. In the human, it is mapped on Yq11.222, encoding one human H-Y (histocompatibility Y) epitope called H-Y/HLA-B7, and is proven to be Y-linked in many species [19]. In DBS, we also showed significantly higher *KDM5D* expression in our male samples as compared to our female samples (*p* < 0.0036). *LOC729444* is an uncharacterized gene with currently unknown functions. It can be found in the cDNA libraries from testis according to the Cancer Genome Anatomy Project (http://cgap.nci.nih.gov/Genes). In our study, we also found *LOC729444* highly expressed in males compared to females (*p* < 0.042). In addition, we found the expression of *TTTY21*, a non-protein coding RNA mapped on Yp11.2, significantly lower in females than in males (*p* < 0.042) from our DBS samples. We also examined the expression of these genes in fresh blood (male *n* = 102, female *n* = 36) and frozen tissues (male *n* = 39, female *n* = 36) that are available from the public domain. We and others found the expression of *XIST* significantly higher in females compared with males in fresh blood samples [20]. Generally, a higher intensity and fold-change are also observed in the fresh samples: 4 fold change in *XIST* (*p* < 4.7 × 10^−20^) and 3 fold change in *KDM5D* (*p* < 2.8 × 10^−23^), while *LOC72944* and *TTTY21* showed higher intensities but no significant fold-change (*p* < 0.022 and *p* < 0.144, respectively). Sex-specific genes including *XIST* and *KDM5D* were also reported to be differentially-expressed in fresh frozen male and female human brains [21]. Thus, although the overall intensity/fold-change is higher in fresh samples, gene expression changes of sex-specific transcripts in DBS are in concordance with those from fresh blood and frozen tissue samples in general. High-quality RNA extracted from matched whole blood at the time when DBS was collected could be used as a reference but unfortunately such samples are difficult, if not impossible to obtain, especially for very old DBS samples.

Overall, we showed that the expression of sex-related genes can be detected, measured, quantified, and correlated with their corresponding expression in each gender. The observed RNA gene expression and gender status preservation in DBS is confirmed by qRT-PCR. Preservation of gender status in DBS is both evidence of and an internal control for accurate gene expression related to certain traits that are preserved in DBS. Consequently, gene expression profiling can be performed to obtain genetic signatures to answer biological questions related to traits of interest.

## 3. Experimental Section

### 3.1. Archived Dried Blood Spots (DBS)

Archived dried blood spots (DBS) were obtained with parental consents from the Michigan Neonatal Biobank (MNB) which stores and manages Michigan’s four million residual newborn dried blood spot cards (http://mnbb.org/). DBS from 53 pairs of cerebral palsy/healthy control subjects (75 male and 31 female) that had been archived from 2.5–15.6 years at ambient temperature were sampled for a large molecular epidemiological study of cerebral palsy using microarray technology. This study was approved by the Institutional Review Boards of Michigan State University, the Michigan Department of Community Health, and the Van Andel Research Institute.

### 3.2. RNA and cDNA Preparation

Total RNA was prepared according to our modified protocol [15]. In brief, three 3 mm punches from each blood spot were homogenized before total RNA extraction using the illustra RNAspin Mini RNA Isolation Kit (GE Healthcare, Buckinghamshire, UK). Total RNA was then concentrated with an RNA Clean & Concentrator-5 Kit (Zymo Research, Orange, CA, USA). RNA quality and quantity were evaluated using an RNA Pico Lab Chip on the Agilent BioAnalyzer (Agilent Technologies, Santa Clara, CA, USA). The average RIN (RNA Integrity Number) was 2.3 ± 0.71. The WT-Ovation Pico RNA Amplification System (NuGEN Technologies, San Carlos, CA, USA) based on the Ribo-SPIA technology was used to amplify total RNA and synthesize first and second-stranded cDNA [22]. Unlike most standard amplification approaches which are based on T7 polymerase cRNA synthesis, this amplification system is initiated both at the 3′ end and randomly throughout the whole transcriptome. Hence, it has the advantage of amplifying non-poly A transcripts and compromised RNA from samples such as archived blood spots in our study.

### 3.3. Labeling, Hybridization, and Scanning Procedures

cDNA was labeled with Alexa Fluor 3 fluorescent dye from the BioPrime Total Genomic Labeling System (Invitrogen Life Technologies, Carlsbad, CA, USA) before purification using the PureLink PCR Purification System (Invitrogen). Purified labeled product was then applied onto a 8 × 60 K whole human genome gene expression microarray (Agilent) for hybridization using standard Agilent procedures. After hybridization for 17 h at 65 °C and 10 rpm rotation speed, the arrays were washed for 2 min each with wash buffer 1 and 2 and scanned with an Agilent G3 high-resolution scanner. Probe features were extracted from the microarray scan data using Feature Extraction software v. 10.7.3.1 (Agilent).

### 3.4. Gene Expression Microarray and Statistical Analyses

All procedures for data processing and analysis were done in R version 2.13.2. Poor-quality signals on the Agilent microarrays were removed when the gProcessed signal was less than two times the gProcessed signal error [23]. Data were normalized between arrays using quantile normalization method [24–26]. Normalized data were then aggregated to gene level to assess differential expression of all individual genes between males and females by moderate *t*-statistic from linear model and empirical Bayes method implemented in the *limma* R package [25,26]. Significance of genes were adjusted for multiple testing using the Benjamini and Hochberg method [27]. Statistical significance was defined when adjusted two-sided *p* ≤ 0.05. The median average of the expression values from the females *g*(f) and males *g*(m) for each gene were computed to generate the heatmap. The group-wise average, [*g*(f) + *g*(m)]/2, was subtracted from each expression value to generate relative expression values for plotting.

### 3.5. Quantitative Real-Time PCR Analysis (qRT-PCR)

To validate the microarray results, the most significantly differentially-expressed sex-specific gene, *XIST*, was evaluated using qRT-PCR using Hs01079824_m1 primer/probe set (Applied Biosystems, Carlsbad, CA, USA). In brief, 20 ng cDNA was mixed with Taqman PCR Super Mix (Applied Biosystems, Carlsbad, CA, USA) and gene-specific forward/reverse primers and probes before subjected to real-time PCR quantification using the ABI PRISM 7500 Fast Real-time PCR System (Applied Biosystems, Carlsbad, CA, USA). The data are presented as number of cycles to threshold.

## 4. Conclusions

In conclusion, we showed the evidence of RNA gene expression preservation and the technical feasibility of obtaining differentially-expressed genes in DBS. Since DBS represents a unique but underutilized research resource for molecular genomic studies, our study provides an important experimental technical advancement to facilitate exploration of DBS in molecular pathogenesis. The capability to obtain molecular genetic profiles (sets of differentially-expressed genes related to certain traits) from DBS offers the best possible baseline picture of a studied subject and can be a powerful molecular tool in allowing us to know possible disease etiology without the interference of post-natal factors such as environment or diet.

## Figures and Tables

**Table 1:** Four differentially-expressed genes that were statistically significantly different between males and females.

Gene ID	Average intensity in males (mean log_2_)	Average intensity in females (mean log_2_)	Adjusted *p* value
*XIST*	5.73	6.92	1.6 × 10^−16^***
*KDM5D*	7.64	6.54	0.0036 **
*LOC729444*	7.99	6.86	0.042 *
*TTTY21*	6.66	5.65	0.042 *

* *p* ≤ 0.05;

** *p* ≤ 0.005;

*** *p* ≤ 0.0001.
